# OPTIFARM: Benchmarking YOLO Architectures for Location-Robust Potato Quality Detection

**DOI:** 10.3390/foods15122121

**Published:** 2026-06-12

**Authors:** Tadej Peršak, Marko Simonič, Jernej Hernavs, Mirko Ficko, Simon Klančnik

**Affiliations:** Faculty of Mechanical Engineering, University of Maribor, Smetanova 17, SI-2000 Maribor, Slovenia

**Keywords:** potato sorting, object detection, YOLO, cross-location generalization, food quality and safety, intelligent inspection, non-destructive testing

## Abstract

Potato sorting in post-harvest processing relies heavily on manual visual inspection, which is physically demanding, subjective, and insufficiently scalable for modern packing lines. This study investigates the feasibility of a low-cost RGB-based optical inspection system for automated potato quality detection using deep learning-based object detection. A controlled imaging platform was constructed using commodity hardware, and a dataset of 19,805 manually annotated instances across 1361 images was collected from two geographically distinct farm locations in Slovenia. A systematic benchmark of 25 model configurations spanning five YOLO architecture families—YOLOv8, YOLOv9, YOLOv10, YOLOv11, and YOLO26—was conducted across three practical quality classes (Edible, Feed, Rotten) using a strict cross-location evaluation protocol in which models were trained on one location and tested on a completely unseen second location. All models achieved strong in-distribution performance (F1 ≥ 0.906), but showed considerable variation under cross-location conditions, with external F1 ranging from 0.792 to 0.918. The yolo26_l configuration achieved the best cross-location performance (F1 = 0.918, mAP@0.5:0.95 = 0.816, ΔF1 = 0.029), demonstrating that transferable representations are achievable under a standard supervised training protocol. Per-class analysis identified feed detection as the primary generalization bottleneck. The results confirm that affordable RGB-based sorting systems are technically feasible and highlight cross-location evaluation as an essential protocol for assessing real-world deployment readiness.

## 1. Introduction

Potatoes (*Solanum tuberosum* L.) are among the most important staple crops worldwide, with global production exceeding 370 million tons annually [1]. Due to their high caloric yield per hectare and adaptability to diverse climatic conditions, potatoes play a crucial role in global food security and agricultural economies [2]. In Slovenia, potato production averages approximately 90,000–100,000 tons annually and is cultivated on roughly 2500–3000 hectares of agricultural land [3]. Despite their economic importance, significant losses occur during post-harvest handling and grading processes [4,5].

A substantial proportion of these losses arises during sorting operations, where potatoes are typically graded according to external quality characteristics [6]. Traditional sorting systems rely on manual visual inspection, where workers classify tubers into categories such as marketable, feed-grade, or rotten. Manual inspection is physically demanding, subjective, and prone to inconsistencies caused by operator fatigue and variability in human perception [7,8]. Modern packing lines often require inspection rates of several potatoes per second, which further increases the likelihood of classification errors in manual systems [9].

To address these limitations, industrial optical sorting systems have been developed using advanced sensing technologies. Commercial solutions from manufacturers such as TOMRA, Rayner, and Newtec integrate multispectral imaging, near-infrared sensors, and X-ray inspection with pneumatic ejection mechanisms to perform high-speed automated sorting [10,11]. These systems can process more than ten potatoes per second while maintaining high detection accuracy. However, their acquisition and installation costs are substantial, limiting accessibility for smaller agricultural producers [9,11].

Recent advances in computer vision and artificial intelligence have created new opportunities for developing low-cost automated inspection systems based on standard RGB imaging hardware [7,12]. Early machine vision approaches for agricultural product inspection relied on handcrafted features such as color histograms, texture descriptors, and threshold-based segmentation algorithms [13]. Although these approaches achieved promising results in controlled laboratory environments, their robustness proved limited when applied to real-world agricultural conditions characterized by illumination variability, soil contamination, and irregular object shapes [14].

The introduction of deep convolutional neural networks (CNNs) significantly improved the performance of computer vision systems by enabling hierarchical features to learn directly from image data. Deep learning architectures have since been successfully applied to numerous agricultural inspection tasks, including fruit grading, plant disease detection, and crop yield estimation [15,16]. Among modern object detection architectures, models from the YOLO (You Only Look Once) family have become particularly popular for real-time industrial applications due to their ability to combine high detection accuracy with low inference latency [16,17].

Several studies have explored the application of YOLO-based architecture for potato quality inspection. Li et al. proposed an improved YOLOv5-based approach incorporating coordinate attention mechanisms for detecting surface defects in potatoes, achieving improved detection performance under controlled imaging conditions [18]. Li et al. developed a YOLOv8-based multi-task system capable of simultaneously detecting potato edibility and segmenting multiple surface defect types with high accuracy [19]. Other studies have investigated deep convolutional networks for detecting potato surface defects and internal disorders such as greening or hollow heart [20,21].

Despite these advances, a critical limitation remains in much of the existing literature. Many studies train and evaluate machine learning models using datasets collected under a single imaging configuration, location, or harvesting batch. Consequently, the generalization capability of these models across different farms, conveyor systems, and seasonal conditions remains insufficiently investigated [12,15,22].

In real agricultural environments, optical inspection systems must operate under varying conditions that introduce significant domain shifts between datasets. These variations may include differences in soil residue on tubers, illumination conditions, camera geometry, potato size distributions, and defect characteristics across different harvest seasons or cultivation regions. Models trained on a single data distribution can therefore experience substantial performance degradation when deployed in new environments, limiting their practical usability [23,24].

From a practical deployment perspective, high detection accuracy achieved on a single dataset is not sufficient to demonstrate the suitability of an optical inspection system for real sorting applications [23,25]. For deployment in agricultural environments, models must remain robust when exposed to previously unseen conditions, including changes in tuber appearance, contamination level, acquisition geometry, and location-specific visual characteristics [26,27]. Recent work in related agricultural domains further confirms that standard mAP values alone are insufficient predictors of field performance and that error-centric reliability metrics evaluated across heterogeneous imaging conditions are more informative for deployment readiness [28]. Therefore, evaluation protocols that explicitly separate training and testing across locations are essential for assessing the true generalization capability of deep learning-based potato inspection systems.

To address these challenges, this study investigates a low-cost optical potato inspection system based on RGB imaging and deep learning. A prototype imaging platform was constructed using commodity hardware consisting of an RGB camera (Basler acA2500-14uc; Basler AG, Ahrensburg, Germany) and uniform LED illumination designed to minimize environmental variability. Using this setup, two location-specific datasets were collected from different farms, enabling evaluation of cross-location model generalization. The resulting dataset contains 1361 annotated images and 19,805 instances, capturing multiple overlapping tubers categorized into three practical classes: Edible, Feed, and Rotten.

Using the collected dataset, a systematic benchmark of several modern YOLO-based detection architectures, including YOLOv8, YOLOv9, YOLOv10, YOLOv11, and YOLO26, was conducted. Models were trained using images from one farm location and evaluated on an unseen dataset from a second location to assess cross-location robustness. Performance was evaluated using standard object detection metrics including Precision, Recall, F1-score, mAP@0.5, and mAP@0.5:0.95, together with inference time measurements relevant for real-time deployment.

The main contributions of this work are summarized as follows:

- Development of a low-cost controlled imaging platform for automated potato inspection, designed to simulate key conditions of industrial sorting systems and suitable for small and medium-scale farms.
- Creation of a two-location annotated potato dataset enabling evaluation of cross-location generalization in agricultural sorting systems.
- Systematic benchmarking of modern YOLO detection architectures for potato quality classification under realistic conveyor conditions.
- Analysis of the trade-off between model size, detection accuracy, and inference speed, providing insights into the deployment of deep learning models for affordable agricultural optical sorting systems.

## 2. Materials and Methods

### 2.1. Study Overview and Experimental Design

The objective of this study was to investigate the feasibility of a low-cost optical inspection system for automated potato quality assessment under conditions relevant to future online sorting. The study focused on the detection of three practically relevant potato classes—Edible, Feed, and Rotten—using RGB image data acquired under controlled illumination conditions and analyzed with deep learning-based object detection models.

The experimental design was specifically structured to evaluate not only detection accuracy under controlled acquisition conditions but also the cross-location generalization capability of the developed models. To achieve this, two independent datasets were collected from different farm locations in Slovenia using the same imaging platform and acquisition protocol. The first dataset, acquired in Kranj, was used for model development, including training and validation. The second dataset, acquired in Gradišče pri Slovenj Gradcu, was reserved exclusively for testing and was not used during model training. This protocol enabled an explicit evaluation of model robustness when transferred to a previously unseen location.

The study followed four main stages. First, a controlled image acquisition platform was developed to simulate key conditions of a future optical sorting system, including standardized RGB imaging, conveyor-based sample presentation, and uniform artificial illumination. Second, annotated datasets were created from the acquired images, with each visible tuber assigned to one of the three target classes. Third, multiple modern YOLO-based detection architectures were trained and validated on the Kranj dataset under identical preprocessing and training settings. Fourth, the trained models were evaluated on the unseen Slovenj Gradec dataset to quantify the performance drop, if any, under cross-location conditions.

The benchmark included several recent YOLO model families and model scales, allowing comparison of lightweight and larger architectures in terms of detection performance and computational efficiency. The evaluation was based on standard object detection metrics, including Precision, Recall, F1-score, mAP@0.5, and mAP@0.5:0.95, complemented by inference time measurements relevant for real-time deployment. In this way, the experimental design supported both methodological comparison and practical assessment of deployment suitability for affordable agricultural sorting systems.

Overall, the study was designed to answer two main research questions:

(1) Can a low-cost RGB-based optical platform provide sufficiently informative image data for reliable potato quality detection?

(2) How well do YOLO-based detection models trained on one farm location generalize to another previously unseen location under realistic agricultural variability?

### 2.2. Data Acquisition System

**Figure 1:**
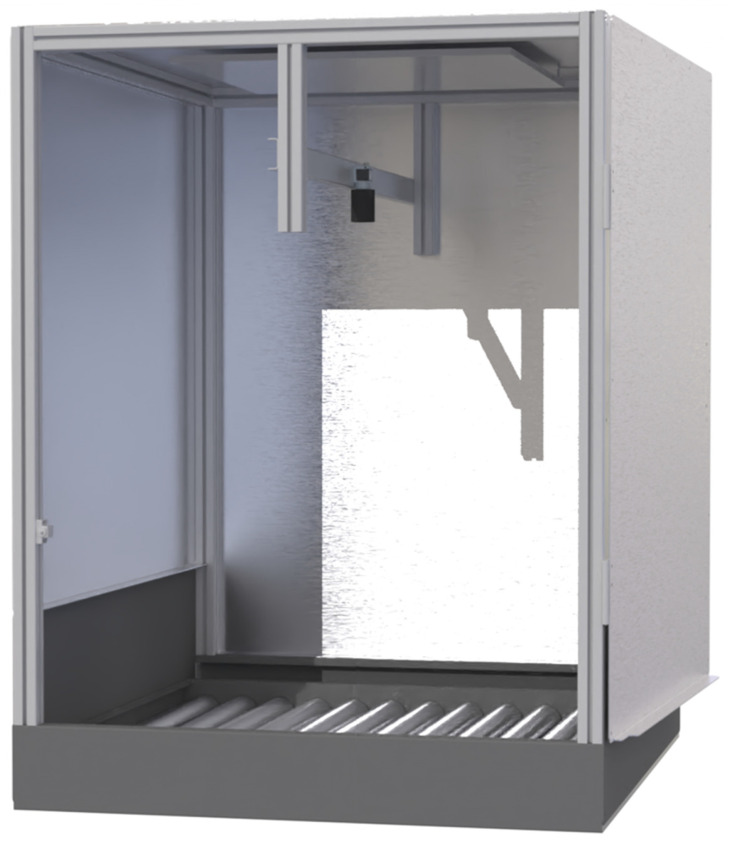
Imaging chamber used for potato data acquisition.

The data acquisition process was performed using a custom-designed imaging chamber, developed to ensure controlled, repeatable, and high-quality image capture conditions. The chamber is constructed from aluminum profiles, while the side panels are made of aluminum sheets, providing a rigid and durable structure and minimizing external light interference. The overall configuration of the imaging system is shown in Figure 1.

The chamber consists of three main components. The lower section incorporates a set of parallel aluminum cylindrical rollers (diameter 60 mm) that serve as a support mechanism for potato samples, ensuring stable positioning and consistent sample orientation during acquisition. The upper section houses an industrial camera (Basler acA2500-14uc; Basler AG, Ahrensburg, Germany) with a 6 mm focal length lens (Basler C125-0618-5M, F1.8; Basler AG, Ahrensburg, Germany) positioned at a fixed distance of 954 mm from the roller surface, ensuring consistent imaging geometry across all samples. Illumination is provided by a square LED panel (MAXXO ZB1224 URG19, 60 × 60 cm, 36 W, 4000 K, 4320 lm; EMOS spol. s r.o., Přerov, Czech Republic) mounted above the camera, whose large surface area minimizes shadows and ensures homogeneous lighting across the field of view.

All images were captured under fixed acquisition parameters to ensure dataset reproducibility. The enclosed chamber design further suppresses ambient light variability, enabling reliable and consistent image acquisition suitable for subsequent detection and classification tasks.

### 2.3. Data Acquisition Software

Image acquisition was controlled through a custom desktop application developed specifically for this study. The application interfaces directly with the industrial camera and provides a unified workflow for image capture and in situ annotation, combining image acquisition and labeling into a single operation and eliminating the need for a separate post hoc annotation stage.

The operator workflow proceeds as follows. Potato samples from different quality classes are placed together on the roller support inside the imaging chamber, forming a dense visual scene representative of online sorting conditions. The operator then captures a single image of the full scene and, within the same application, draws a bounding box around each visible tuber and assigns it to one of the three target classes (Edible, Feed, or Rotten). Once the scene has been fully annotated, the application stores the acquired image together with a corresponding text file containing all class labels and normalized bounding-box coordinates in YOLO format. The potatoes are then rearranged or replaced with new samples, and the procedure is repeated to obtain additional images with varying tuber configurations.

Storing annotations in YOLO format at the time of acquisition ensured direct compatibility with the subsequent training pipeline. All captured images and their associated annotation files were organized by acquisition location and stored on the local workstation for subsequent preprocessing and model training.

### 2.4. Dataset Description

The dataset used in this study was collected at two agricultural locations in Slovenia using the same controlled imaging platform and acquisition protocol. The first dataset was acquired in Kranj and contains 1060 images, while the second dataset was acquired in Gradišče pri Slovenj Gradcu and contains 301 images. Together, these two datasets were used to support both model development and cross-location evaluation. An overview of the two image datasets used in this study is provided in Table 1.

The complete dataset comprises 19,805 manually annotated instances across 1361 images. The distribution of annotated instances across quality classes and acquisition locations is summarized in Table 2. A pronounced class imbalance is present at both locations, with the edible class representing most of the annotated instances (62.0% in Kranj, 84.9% in Slovenj Gradec). The feed and rotten classes together account for 38.0% of Kranj instances but only 15.1% of Slovenj Gradec instances. This difference in class distribution between the two locations constitutes an additional source of domain shift beyond visual appearance variability and is relevant to the interpretation of cross-location classification performance discussed in Section 3.2.

Each image contains multiple potatoes captured simultaneously under controlled illumination conditions. On average, each image includes approximately 12 to 15 tubers, resulting in a dense visual scene with frequent object proximity and partial overlap. This acquisition setup was intentionally designed to approximate the visual complexity expected in future online sorting scenarios rather than isolated single-object inspection.

The dataset exhibits substantial visual variability relevant to automated potato quality assessment. This includes differences in tuber size, shape, and surface appearance, as well as varying degrees of mutual overlap, soil contamination, surface damage, and visible defects. Such variability is important for evaluating the robustness of object detection models under realistic agricultural conditions, where the appearance of potatoes is influenced by harvesting, handling, and field-specific factors.

In addition to inter-object variability within each image, the use of two geographically distinct collection sites introduces location-related variation in the dataset. These differences may include changes in tuber appearance, contamination patterns, and other visual characteristics associated with different production conditions. Furthermore, the difference in class distribution between the two locations—with a notably higher proportion of minority class instances in Kranj than in Slovenj Gradec—reflects real-world variability in potato batch composition and should be considered when interpreting cross-location performance results. For this reason, the dataset is particularly suitable for assessing cross-location generalization, which is one of the central aims of this study.

### 2.5. Class Definitions and Annotation Procedure

For the purposes of this study, each visible potato instance was assigned to one of three target classes: Edible, Feed, or Rotten. The operational definitions of these annotation classes, together with their inclusion criteria, are summarized in Table 3. The Edible class included potatoes with no visually significant defects that would prevent their use for human consumption. The Feed class included potatoes with visible external defects or quality deterioration that reduced their market value for human consumption but did not correspond to severe rot. The Rotten class included potatoes showing clear signs of advanced decay, including extensive discoloration, tissue degradation, or other visually evident symptoms of rot.

**Figure 2:**
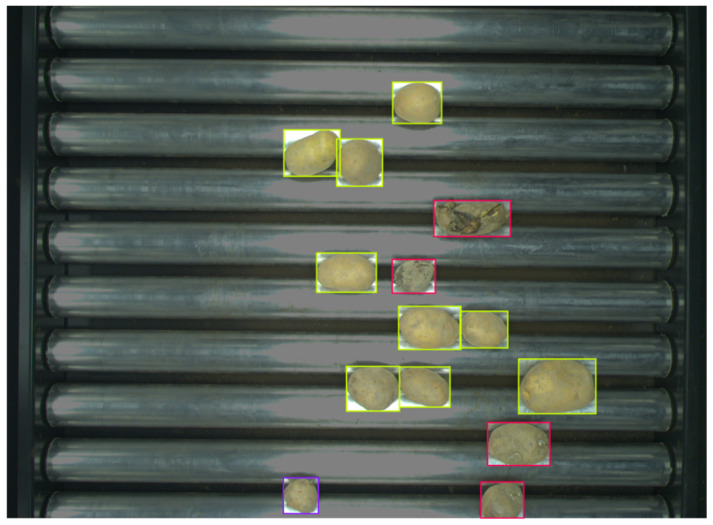
Example of manual bounding-box annotations used in the dataset. Visible potatoes were assigned to one of three classes: Edible (yellow), Feed (purple), or Rotten (red).

To ensure consistency across the dataset, the annotation procedure was standardized and applied identically at both collection locations. All images from both locations were annotated by the same reference labeler using identical class definitions, labeling rules, and bounding-box annotation protocol. As described in Section 2.2, annotations were produced in situ during image acquisition using the custom capture application: immediately after each image was captured, every visible potato was enclosed in a single bounding box and assigned to the most appropriate class based on its dominant visible quality condition. An example of the resulting bounding box is shown in Figure 2.

The YOLO annotation format, in which each labeled object is represented by its class identifier and normalized bounding-box coordinates (width, height), was selected to ensure direct compatibility with the evaluated YOLO-based detection architectures.

Because both datasets were annotated by the same reference labeler using identical class definitions and the same acquisition application, a consistent labeling standard was maintained across the entire dataset. Borderline cases between Edible and Feed (minor surface damage or light discoloration) and between Feed and Rotten (advanced discoloration without full tissue breakdown) were resolved by assigning the class corresponding to the dominant visible quality condition, in accordance with the operational definitions in Table 3. Applying the same decision rules at both locations ensures that any performance differences observed between the internal and external test sets reflect true visual domain shifts rather than inter-labeler variability.

### 2.6. Data Splits and Preprocessing

The dataset acquired in Kranj was used for model development and internal evaluation. It was divided at the image level into training, validation, and internal test subsets using an 80:10:10 split (random, fixed seed). The training subset was used for model fitting, the validation subset for monitoring convergence and model selection during training, and the internal test subset for evaluating performance on previously unseen images originating from the same location and acquisition setup.

Model selection was performed in two stages, both of which were independent of the external test set. First, within each of the 25 training runs, the best checkpoint was selected using the validation mAP@0.5:0.95 metric (metrics/mAP50-95(B)), computed on the held-out Kranj validation subset. Second, the external Slovenj Gradec test set was used exclusively for final evaluation and was never accessed during training, checkpoint selection, or hyperparameter tuning. Consequently, the external performance reported in Section 3 reflects a genuinely held-out assessment. We note that yolo26_l is identified as the strongest cross-location model post hoc—that is, based on its external-test performance reported here—rather than having been pre-selected as a recommended model prior to external evaluation; the distinction between these two roles is discussed in Section 4. The resulting data partitioning is summarized in Table 4.

The dataset acquired in Gradišče pri Slovenj Gradcu was reserved exclusively as an external test set and was not used during training, validation, or internal testing. This design enabled a separate evaluation of cross-location generalization, allowing the comparison of model performance under in-distribution and out-of-distribution conditions.

No offline image preprocessing was applied during dataset preparation: images were kept at their native acquisition resolution (1280 × 932), and no tiling, slicing, cropping, or pre-resizing was performed. All preprocessing was handled on-the-fly by the Ultralytics training pipeline, which resized images to a target size of 1280 pixels along the longer dimension while preserving aspect ratio (rect mode) and normalized pixel values to the range [0, 1]. The complete preprocessing and augmentation configuration is summarized in Table 5.

To improve model robustness while preserving the integrity of dense multi-object scenes, a deliberately conservative augmentation strategy was employed. The selected transformations introduce limited geometric and photometric variability without altering the spatial structure of the scene. More aggressive augmentations commonly used in object detection—such as mosaic composition or large-scale geometric transformations—were intentionally excluded, as they can distort object density, alter relative spatial relationships, and reduce the effective resolution of individual tubers. This is particularly critical in the present setting, where multiple closely packed instances must be detected reliably. By restricting augmentation to mild variations, the training process encourages generalization while maintaining consistency with the real acquisition conditions. Evaluation datasets were kept strictly unaugmented to ensure an unbiased assessment of model performance under both in-distribution and cross-location scenarios.

### 2.7. Detection Models (YOLO Variants) and Training Configurations

To assess the suitability of modern one-stage object detectors for potato quality inspection, a benchmark was conducted using five YOLO model families: YOLOv8, YOLOv9, YOLOv10, YOLOv11, and YOLO26. For each family, the standard scale variants n, s, m, l, and x were evaluated, resulting in a total of 25 model configurations. This design enabled a systematic comparison of lightweight, medium-sized, and larger detection models under a unified experimental protocol.

All models were implemented using the Ultralytics YOLO framework (version 8.4.18; Ultralytics Inc., Frederick, MD, USA) with a PyTorch 2.5.1 backend (torchvision 0.20.1, CUDA 12.1). To ensure a fair comparison, all model configurations were trained and evaluated using the same dataset split, preprocessing pipeline, and evaluation procedure. The common training configuration applied to all evaluated models is summarized in Table 6.

The main training settings included the number of training epochs, batch size, input image size, initial learning rate, optimizer, and augmentation strategy. These parameters were kept as consistent as possible across all experiments to isolate the influence of model family and model scale on detection performance. Model training was performed on a workstation equipped with a dedicated GPU, as specified in Table 6.

Performance was evaluated using standard object detection metrics, including Precision, Recall, F1-score, mAP@0.5, and mAP@0.5:0.95. In addition, inference time was recorded to assess the practical suitability of the evaluated models for future real-time deployment in optical sorting applications.

The evaluation protocol included both internal and cross-location testing. First, the models were assessed on the internal test subset originating from Kranj, which enabled the estimation of performance on previously unseen images acquired under the same location and imaging setup as the training data. Second, the trained models were evaluated on the completely unseen dataset from Gradišče pri Slovenj Gradcu, which served as an external test set for assessing cross-location robustness and sensitivity to location-related visual variability.

### 2.8. Non-YOLO Baseline

To provide a comparison point outside the YOLO family, a Faster R-CNN detector with a ResNet-50 FPN backbone (COCO-pretrained) was fine-tuned on the identical training split. The classification head was replaced to match the three potato quality classes plus background. Training used SGD (learning rate 0.005, momentum 0.9, weight decay 0.0005), a StepLR scheduler (step size 3, gamma 0.1), batch size 4, and early stopping with patience 5 based on validation mAP@0.5:0.95; training converged at epoch 12 with the best checkpoint at epoch 7. Input images were resized to a longest-side dimension of 1280 px with preserved aspect ratio, matching the YOLO preprocessing. The model was evaluated on the same internal and external test sets using identical metrics.

## 3. Results

### 3.1. Overall Detection Performance Across 25 YOLO Configurations

Table 7 summarizes the detection performance of all 25 YOLO configurations on both test sets, together with the generalization drop (ΔF1, ΔmAP@0.5:0.95) from internal to external. On the internal Kranj test set (106 images), all models achieved F1 ≥ 0.906 and mAP@0.5:0.95 ≥ 0.863, indicating that every family and scale could learn the task under the in-domain random split. The highest internal F1 was obtained by yolo11_s (0.9517), while the highest internal mAP@0.5:0.95 was obtained by yolo26_l (0.8895); in total, eleven of the twenty-five configurations—including yolo26_l (internal F1 = 0.9464)—exceeded an internal F1 of 0.940. Compact scales (n, s) were competitive with larger scales on this split, consistent with the limited visual variability of a single-location random split. These small between-model margins are a known property of in-domain random splits and do not, on their own, establish which model should be deployed; the more informative signal is how the ranking changes when the test distribution shifts (Section 3.2).

### 3.2. Per-Class Detection Performance

Aggregate metrics conceal pronounced differences between the three potato classes. Table 8 reports per-class F1 and AP@0.5:0.95 for yolo26_l—the best external-generalization model identified in Section 3.1—on both the internal Kranj and the external Slovenj Gradec test sets, together with the per-class ΔF1.

The majority class is edible, which accounts for 3819 of 4495 external instances (85.0%). It generalizes essentially without loss: internal F1 0.9640 vs. external F1 0.9618 (ΔF1 = 0.0022). Moreover, external AP@0.5:0.95 exceeds the internal value (0.9848 vs. 0.9239). For this class, the model has saturated the task across both locations.

The two minority classes remain the bottleneck, but in distinct ways. Feed suffers the largest per-class generalization drop (ΔF1 = 0.2287, external F1 = 0.6518). Breaking this down by error mode, feed precision falls sharply from 0.8988 on the internal set to 0.5552 on the external set, while recall falls only mildly (0.8629 to 0.7892). The absolute error counts make the mechanism explicit: false positives grow from 34 to 270, whereas false negatives grow from 48 to 90. The dominant failure on the external set is therefore misclassification of edible tubers as feed rather than missed feed detections. Rotten shows the opposite pattern: external precision holds up (0.8517) while recall drops from 0.9815 on the internal set to 0.7149 on the external set (false negatives grow from 5 to 71); a non-trivial fraction of rotten potatoes is simply missed.

Two factors plausibly drive this pattern. First, class imbalance: feed and rotten together represent only 15% of the external instances, so each class contributes less supervision signal during training, and small calibration errors translate into many false positives when those classes are evaluated at location-shifted distributions. Second, visual ambiguity: the boundary between edible and feed is defined by cosmetic and size criteria that can vary between grading standards and between acquisition sites, while rotten defects are often localized to a small region of a tuber and are easy to miss when the defect faces away from the camera.

**Figure 3:**
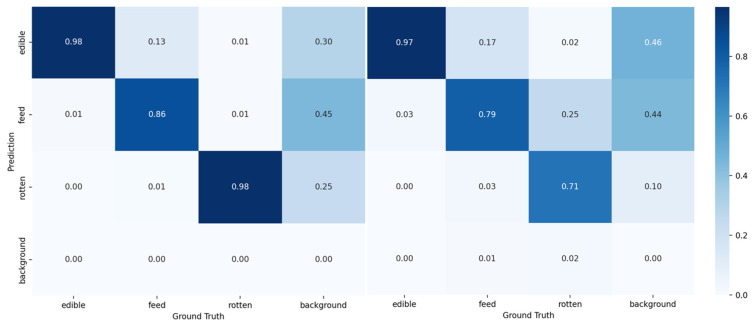
Confusion matrices for the yolo26_l model in internal (**left**) and external (**right**) test sets.

The class-wise behavior of the model is further illustrated using confusion matrices. Figure 3 presents the confusion matrices for the yolo26_l model on both the internal (left) Kranj test set and the external (right) Slovenj Gradec test set, providing a detailed view of classification performance and error distribution across the three classes.

The internal confusion matrix (left) shows strong diagonal dominance across all classes, indicating high classification accuracy under in-distribution conditions. The edible and rotten classes are classified with near-perfect accuracy, while the feed class also demonstrates strong performance with limited misclassification.

In contrast, the external confusion matrix (right) reveals a noticeable increase in off-diagonal elements. The most prominent change is the increased confusion between edible and feed classes, where a substantial proportion of edible instances are misclassified as feed. This directly corresponds to the observed drop in feed precision reported in Table 8. Additionally, the external matrix shows reduced recall for the rotten class, reflected in an increased number of missed rotten instances.

Overall, the confusion matrices confirm that while object localization remains stable across locations, classification performance—particularly for minority classes—is more sensitive to domain shift.

### 3.3. Qualitative Detection Results

To complement the quantitative evaluation, qualitative detection results were inspected for the best-performing model, yolo26_l, on both the internal Kranj test set and the external Slovenj Gradec test set. The aim of this analysis was to visually assess whether the detector correctly localized individual tubers in dense scenes and whether class predictions remained stable under cross-location visual variability.

#### 3.3.1. Robust Detections in Controlled Conditions

**Figure 4:**
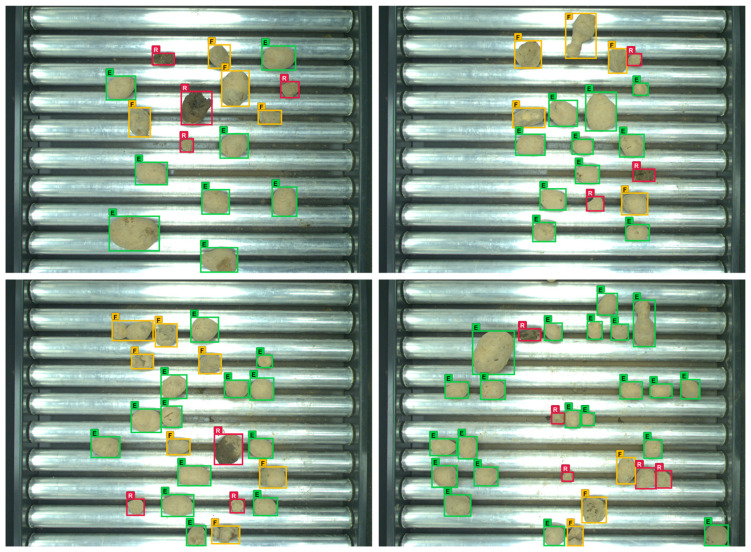
Successful detections under controlled internal test conditions. Class color-coding: E (green) = edible, F (yellow) = feed, R (red) = rotten.

Qualitative inspection of detection results on the internal Kranj test set indicates that the yolo26_l model is capable of accurately localizing and classifying individual potato instances under controlled acquisition conditions, as shown in Figure 4. The detector consistently identifies tubers of varying sizes and shapes, with bounding boxes well aligned to object boundaries. Class predictions are generally consistent with the ground truth annotations, particularly for the majority edible class, while feed and rotten instances are also correctly identified when visual characteristics are clearly expressed. These observations are consistent with the strong internal performance reported in Section 3.2, confirming reliable model behavior under in-distribution conditions.

Representative qualitative detection results of yolo26_l on the internal Kranj test set. Bounding boxes are color-coded by class: E (green), F (yellow), and R (red) denote edible, feed, and rotten potatoes, respectively. The presented examples illustrate accurate localization and consistent classification across multiple instances, without observable false positives or missed detections.

#### 3.3.2. Robust Detections Under Cross-Location Conditions

**Figure 5:**
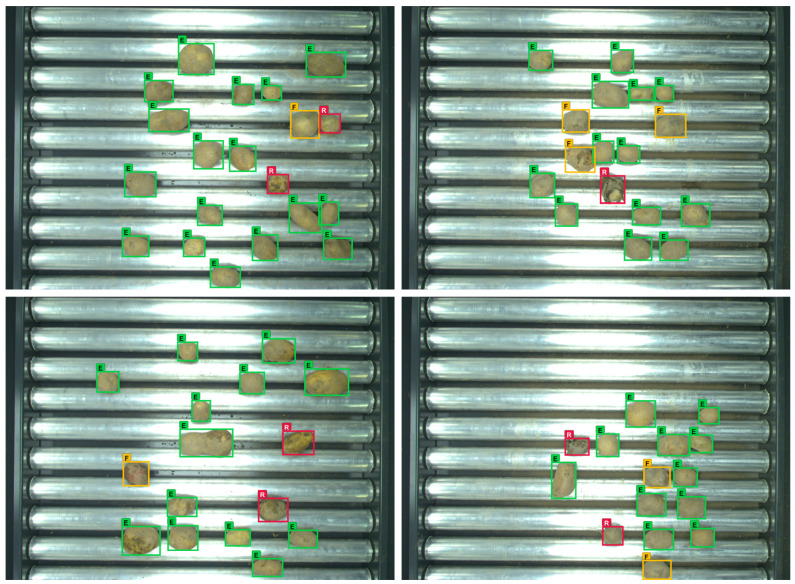
Qualitative cross-location performance of yolo26_l on the external Slovenj Gradec test set, illustrated on four representative scenes. Class color-coding: E (green) = edible, F (yellow) = feed, R (red) = rotten.

To further assess model robustness, qualitative detection results were inspected on the external Slovenj Gradec test set, which introduces variations in tuber appearance compared to the training data. As shown in Figure 5, the yolo26_l model maintains stable localization performance, with bounding boxes generally well aligned to individual potato instances across different scenes.

Across the four example scenes, detection of the majority edible class remains consistent, reflecting the minimal performance drop reported in Section 3.2. The model correctly identifies tubers despite variations in surface texture, contamination, and color, indicating that it does not rely solely on location-specific visual features learned during training.

However, qualitative differences in classification behavior can be observed in more visually ambiguous cases, particularly between edible and feed classes. While many instances are still correctly classified, some predictions appear less distinct compared to the internal test set, which is consistent with the observed decrease in precision for the feed class. Overall, the results confirm that the model generalizes well at the object detection level, while class-level ambiguity remains present under cross-location variability.

#### 3.3.3. Failure Cases

**Figure 6:**
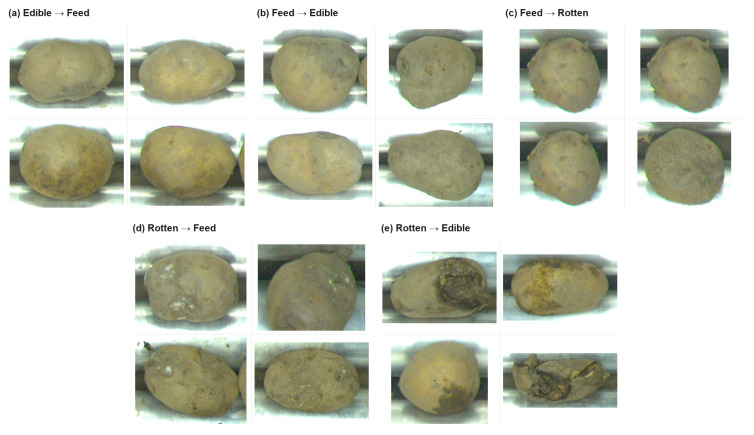
Representative failure cases of the yolo26_l model on the external Slovenj Gradec test set.

Qualitative inspection of failure cases was conducted to better understand the error patterns identified in the quantitative evaluation (Section 3.2) and confusion matrices. The analysis focuses on typical misclassification scenarios observed on the external test set, highlighting the visual characteristics that contribute to incorrect predictions. Representative examples of each misclassification type are shown in Figure 6.

Edible misclassified as Feed (a). The most frequently observed failure mode on the external test set corresponds to edible potatoes being incorrectly assigned to the feed class, which directly explains the sharp drop in feed precision from 0.8988 to 0.5552. In the presented examples, both misclassified tubers exhibit smooth, undamaged surfaces with no visible quality defects. The most plausible trigger is subtle variation in skin tone or surface coloration—slight yellowish or uneven hues—that the model learned to associate with cosmetic degradation during training on the Kranj dataset. This suggests that the model acquired a color-biased decision boundary that does not generalize well to the different soil residue and tuber pigmentation characteristics of the Slovenj Gradec location.

Feed is misclassified as Edible (b). In these cases, the model fails to detect actual quality defects and assigns a higher-quality label. The presented examples include tubers with small dark spots, minor surface lesions, or irregular shape deformation that are consistent with the feed class definition. When defects are spatially localized, low in contrast relative to the surrounding skin, or confined to a small fraction of the visible surface area, the model appears unable to accumulate sufficient evidence to override the dominant edible prediction. This behavior is consistent with the relatively mild recall drop for the feed class observed in Section 3.2.

Feed is misclassified as Rotten (c). A less frequent but practically relevant error involves feed-grade potatoes being over-classified as rotten. The examples show tubers with dark surface patches or localized discoloration that do not represent advanced tissue decay but exhibit textural and chromatic features that partially overlap with early-stage rot symptoms. This confusion is inherent to the visual ambiguity between the upper boundary of the feed class and the lower boundary of the rotten class, as defined in Table 3, and is exacerbated under cross-location conditions where the exact appearance of these borderline cases differs from the training distribution.

Rotten is misclassified as Feed (d). These examples illustrate cases where the model detects a quality problem but underestimates its severity. One case shows a tuber with white surface deposits consistent with mold growth on an otherwise structurally intact surface; the model appears not to associate this specific symptom with the rotten class. A second example presents a tuber where decay is spatially limited to a small surface region, potentially on a partially occluded or non-camera-facing side. Both cases highlight a fundamental limitation of single-view RGB acquisition: defects confined to less visible areas reduce the discriminative signal available to the detector, regardless of model capacity.

Rotten is misclassified as Edible (e). The most critical failure mode from a practical sorting perspective involves severely degraded tubers being assigned to the edible class. In the presented examples, one tuber displays a clearly visible dark cavity with advanced tissue breakdown occupying a substantial portion of its surface, yet the surrounding area remains visually intact. Another example shows an almost entirely collapsed and shriveled tuber with extensive blackening. These cases likely correspond to rare visual patterns that were underrepresented in the Kranj training set, resulting in insufficient model calibration for their detection on the external dataset. From a deployment perspective, this error type carries the highest operational risk, as rotten potatoes passed as edible would directly compromise product quality downstream.

Taken together, the failure cases confirm that the primary challenge is not general object localization, which remained stable across locations, but fine-grained quality discrimination under distribution shift. The dominant error mechanism is the confusion between visually adjacent classes—edible and feed on one end, and feed and rotten on the other—amplified by location-specific differences in tuber appearance, contamination, and defect expression.

### 3.4. Error Analysis

To complement the aggregate performance metrics reported in Section 3.1, a detailed error analysis was performed for all 25 YOLO configurations by examining absolute false positive and false negative counts on both the internal Kranj and external Slovenj Gradec test sets. The complete per-model error counts are summarized in Table 9.

Across all models, the transition from internal to external evaluation produced a consistent and substantial increase in both FP and FN counts. On the internal set, FP counts across all 25 configurations range from 90 to 204 (median 120), corresponding to per-image rates of 0.85 to 1.92 FP/image. On the external set, FP counts increased to a range of 462 to 1174, with per-image rates of 1.53 to 3.90—representing an average increase of approximately 2.5× relative to internal conditions. This consistent amplification of false positives confirms that domain shift primarily manifests as excess spurious detections rather than missed objects, consistent with the feed precision collapse described in Section 3.2.

False negative counts showed a more moderate but still substantial increase, rising from an internal range of 67–115 (0.63–1.08 FN/img) to an external range of 293–792 (0.97–2.63 FN/img). The relative FN increase was smaller than the FP increase, indicating that object localization partially held up under domain shift, while classification confidence was more severely disrupted.

The best external-generalization model, yolo26_l, was the clear outlier in this comparison. It recorded the lowest external FP count of all 25 configurations (462 FP, 1.53 FP/img) and the lowest external FN count (293 FN, 0.97 FN/img). Notably, yolo26_l was the only model to achieve fewer than one false negative per image on the external set, and its external FP/img rate of 1.53 was more than 2× lower than the worst-performing models (yolov9_n: 3.82, yolov9_x: 3.90). This error profile directly explains its superior external F1 and mAP@0.5:0.95 scores reported in Table 7.

### 3.5. Inference Speed

To assess the practical suitability of the evaluated models for future real-time deployment in optical sorting applications, inference latency and throughput were measured as GPU compute (forward pass plus NMS, excluding disk I/O). Results are summarized in Table 10.

Inference latency ranged from 18.8 ms/image (yolov10_n, 53.1 FPS) to 48.4 ms/image (yolov9_x, 20.6 FPS). Compact n-scale models consistently achieved the lowest latency. The yolo26_l model achieved an inference latency of 42.2 ms/image (23.7 FPS), corresponding to approximately 320 potato classifications per second at an assumed density of 13.5 tubers per image. While lower than the most compact configurations, this remains within the throughput range relevant for small- and medium-scale sorting systems. These figures reflect GPU compute only; in a deployed system, images would be acquired through direct camera-to-memory transfer rather than disk loading, so end-to-end throughput will additionally depend on the camera interface and host pipeline.

To verify that no content-dependent latency difference exists between the two test sets—as expected, since the forward pass operates on fixed-size inputs independent of image content—a segmented profiling analysis was performed, separately timing preprocessing, the forward pass (backbone + neck + head), and NMS post-processing, with GPU synchronization at each stage boundary and warmup iterations excluded (Table 11). Averaged across all 25 models, forward pass time was 49.2 ms on the internal set versus 48.7 ms on the external set, and NMS time was 1.00 ms versus 1.04 ms—differences within measurement noise. This confirms that inference latency is determined by model architecture and input resolution, not by scene content or object density.

Forward pass and NMS are content-independent. Preprocessing is dominated by disk image loading and is not representative of camera-based deployment.

A secondary observation concerns the relationship between NMS cost and detection-head design. The number of raw candidate boxes generated before NMS differed sharply between architecture families: the dense heads of YOLOv8, YOLOv9, and YOLOv11 produced approximately 33,600 raw boxes per image, whereas the NMS-light designs of YOLOv10 and YOLO26 produced approximately 300. This is reflected in NMS time, which averaged 1.56 ms for the high-box-count families versus 0.26 ms for YOLOv10/YOLO26. NMS cost is therefore associated with the number of candidate boxes produced by each architecture, not with test-set image content.

From a deployment perspective, the results confirm that compact models (n, s scale) offer the best throughput characteristics, while larger models provide superior detection robustness at the cost of increased inference time. The yolo26_l model offers a favorable accuracy-speed trade-off for systems where detection reliability across locations is prioritized over maximum throughput.

### 3.6. Comparison with a Non-YOLO Baseline

To verify that the YOLO results are competitive against a detector from a different architectural family, a Faster R-CNN ResNet-50 FPN model was trained and evaluated under identical conditions (Table 12). On the internal Kranj test set, Faster R-CNN achieved an F1 of 0.9211 (mAP@0.5:0.95 = 0.8544). On the external Slovenj Gradec test set, it achieved an F1 of 0.8805 (mAP@0.5:0.95 = 0.7876). This places Faster R-CNN close to the second tier of YOLO configurations on external F1 (comparable to yolov9_l at 0.8815), but below the best YOLO model, yolo26_l (external F1 = 0.9176). The non-YOLO baseline therefore confirms that the strongest YOLO configuration retains an advantage under cross-location conditions, while also demonstrating that the cross-location detection task is tractable across detector families rather than being specific to YOLO.

### 3.7. Statistical Comparison and Training Convergence

The benchmark in Section 3.1 reports a single training run per configuration. To assess whether the external-test superiority of yolo26_l reflects a genuine performance difference rather than chance variation, two complementary analyses were performed: bootstrap confidence intervals and pairwise significance testing on external per-image F1, together with an examination of training convergence behavior.

Per-image F1 scores were computed for all 25 models on the 301 external test images, and 95% confidence intervals were estimated using bootstrap resampling (10,000 resamples). The yolo26_l model achieved the highest mean per-image external F1 (0.9318, 95% CI [0.9244, 0.9390]), and its confidence interval did not overlap with that of any other configuration. The five top-ranked configurations are reported in Table 13.

Note that the mean per-image F1 reported here differs slightly from the aggregate external F1 in Table 7 (0.9176 for yolo26_l), as the former averages the F1 score computed independently on each image, whereas the latter pools all detections across the test set before computing a single F1; both are standard and report the same underlying performance from complementary perspectives.

Pairwise Wilcoxon signed-rank tests were performed comparing yolo26_l against each of the other 24 models on paired per-image F1, with Holm correction for multiple comparisons. All 24 comparisons were statistically significant after correction (all Holm-adjusted *p* < 10^−13^). The smallest margin was against yolov9_l (mean per-image difference 0.0350, Holm-adjusted *p* = 8.3 × 10^−14^). These results indicate that the external superiority of yolo26_l over the other YOLO configurations is statistically robust at the image level.

**Figure 7:**
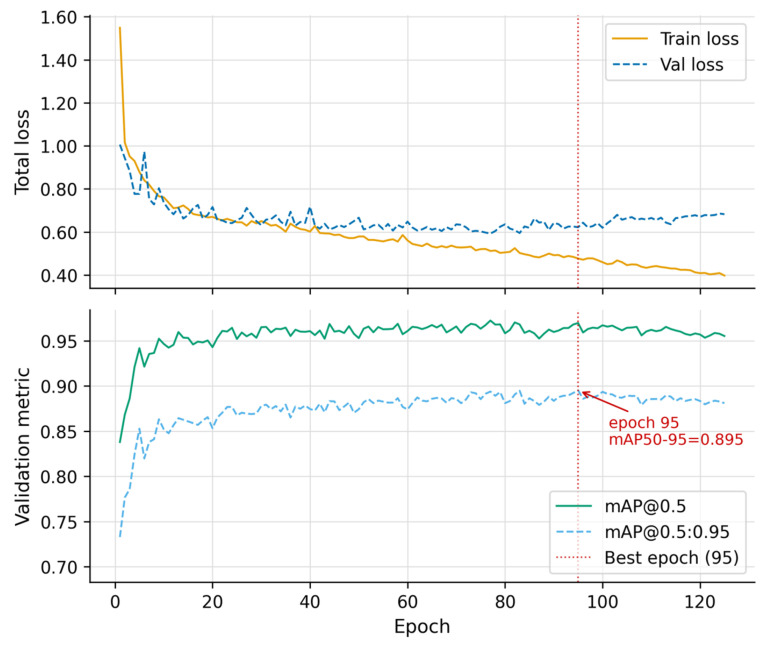
YOLO26-L training convergence: loss (**top**) and validation mAP (**bottom**). Best checkpoint at epoch 95.

**Figure 8:**
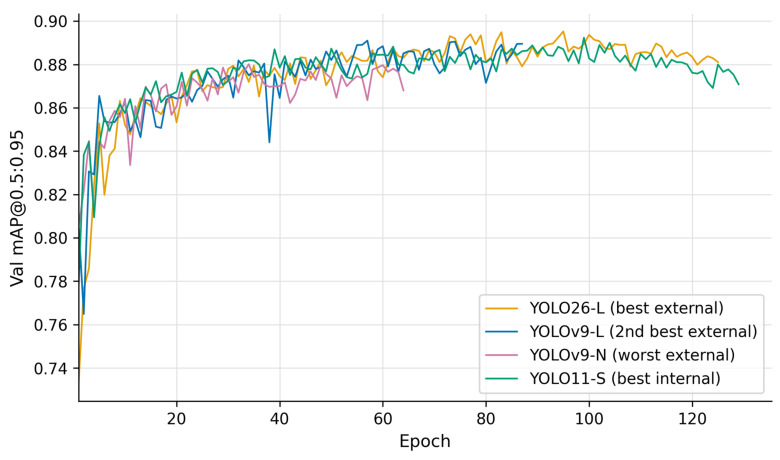
Validation mAP@0.5:0.95 convergence for four representative configurations.

Training convergence behaviour is shown in Figure 7 and Figure 8. For the best-performing model (Figure 7), training and validation loss decreased rapidly within the first 20 epochs, after which validation loss exhibited a mild upward drift while validation mAP remained stable; the best checkpoint (epoch 95, validation mAP@0.5:0.95 = 0.895) was selected by the validation mAP@0.5:0.95 criterion, confirming that checkpoint selection rather than final-epoch weights was the appropriate choice. Across the four representative configurations spanning the observed performance range (Figure 8), all models reached a comparable validation plateau (mAP@0.5:0.95 ≈ 0.88–0.90) within approximately 40 epochs, despite their substantially different external-test generalization. This reinforces the central finding that internal validation performance alone is not a reliable predictor of cross-location robustness. Complete per-epoch convergence data for all 25 configurations are provided as Supplementary Materials.

## 4. Discussion

The following discussion interprets the experimental results in the context of cross-location deployment robustness, examines class-level error patterns, and situates the findings within the broader landscape of deep learning-based potato inspection research, with the aim of identifying both the practical potential and the current limitations of the proposed system.

### 4.1. Interpretation of Detection Performance

The uniformly high internal performance observed across all 25 evaluated configurations—with every model achieving F1 ≥ 0.906 and mAP@0.5:0.95 ≥ 0.863 on the internal Kranj test set—reflects a fundamental property of single-location random splits rather than a reliable indicator of model quality. When training and test images originate from the same acquisition session and location, the visual distribution is effectively identical, and even compact models with limited representational capacity can saturate the task. This result is consistent with the broader pattern observed in agricultural computer vision, where in-domain benchmarks frequently overestimate practical deployment performance [23,25]. The key diagnostic signal is therefore not the absolute internal score, but the magnitude and consistency of the performance drop when the test distribution shifts to a new location.

Interpreted through this lens, the external Slovenj Gradec results reveal a substantially more differentiated model landscape than the internal results alone would suggest. Models that ranked closely on the internal set diverged considerably under domain shift, with external F1 values ranging from 0.792 (yolov9_n) to 0.918 (yolo26_l)—a spread more than five times wider than on the internal set. This confirms that cross-location evaluation is a more informative selection criterion for deployment-oriented benchmarking and that internal performance alone is insufficient to identify robust models. This observation aligns with recent findings in related agricultural domains, where variance-based reliability metrics evaluated across heterogeneous imaging conditions were shown to be more predictive of field performance than absolute mAP values [28].

A note on model selection is warranted here. Under a strict a priori criterion based solely on internal (Kranj) test F1, the top-ranked configuration would be yolo11_s (internal F1 = 0.9517), with yolo26_l ranking third internally (F1 = 0.9464). The identification of yolo26_l as the strongest configuration is therefore based on its external (Slovenj Gradec) performance and is, by construction, a post hoc observation rather than an a priori selection. We report it as such deliberately: the central finding of this study is precisely that internal-test ranking is a poor predictor of cross-location ranking, and that the model best suited for deployment can only be identified once out-of-distribution performance is measured. The external test set was not used to tune any model or hyperparameter; it was used solely to evaluate already-trained models. We therefore do not claim that yolo26_l was selected without reference to external performance—it was not—but rather that its external superiority is a measured outcome on a genuinely held-out location, which is the quantity of interest for deployment readiness.

Among all evaluated configurations, yolo26_l provided the best balance of accuracy and cross-location robustness, achieving the highest external F1 and mAP@0.5:0.95 while also recording the smallest ΔF1 of any model (0.029). Several architectural properties of YOLO26 may plausibly contribute to this result. Unlike previous YOLO generations, YOLO26 replaces Distribution Focal Loss with a simplified bounding box regression formulation and introduces Progressive Loss Balancing (ProgLoss) together with Small-Target-Aware Label Assignment (STAL), which promotes more stable and class-balanced supervision during training [29]. The MuSGD optimizer, a hybrid inspired by recent large model training techniques, further stabilizes convergence. Taken together, these mechanisms may encourage the model to learn more transferable feature representations rather than overfitting to location-specific visual statistics present in the Kranj training set. However, since no ablation study was conducted, this remains a plausible interpretation rather than a confirmed causal explanation, and further experiments isolating individual architectural components would be needed to substantiate it.

A notable finding is that model scale did not reliably predict cross-location robustness. Several x-scale configurations achieved strong internal results but exhibited larger external performance drops than their smaller counterparts, while yolo26_l—a large but not the largest scale—outperformed all x-scale models on the external set. This suggests that generalization under domain shift is not simply a function of model capacity but depends on how well the learned representations transfer across location-specific visual conditions. Similar observations have been reported in the agricultural domain adaptation literature, where model architecture and training strategy were found to be more decisive than parameter count for cross-environment robustness [23,26].

Taken together, the results provide affirmative answers to both research questions posed in Section 2.1: a low-cost RGB imaging platform proved capable of supporting reliable potato quality detection, and YOLO-based models—particularly yolo26_l—demonstrated meaningful cross-location generalization under realistic agricultural variability.

### 4.2. Cross-Location Generalization

Cross-location generalization is the central aspect of this study. The external Slovenj Gradec dataset was not used during training or validation, making it a realistic test of deployment robustness. The observed performance drop between the internal and external datasets confirms the presence of domain shift between the two acquisition locations.

This domain shift likely arises from differences in potato appearance, surface contamination, defect visibility, size distribution, and handling conditions. Even though the same imaging platform and acquisition protocol were used at both locations, biological and production-related variability remained present. This is important because real sorting systems are expected to operate across different farms, batches, seasons, and potato conditions.

The relatively small overall performance drop of yolo26_l indicates that the model generalized well at the object localization level. Bounding boxes remained stable on the external dataset, and the majority of edible class showed almost no degradation. This suggests that the detector learned robust shape and appearance features for normal potatoes.

This localization stability should be interpreted considering the COCO-pretrained initialization used for all models. Given the modest training set of 848 images, the large-scale object priors learned from COCO pretraining likely contributed substantially to the robustness of bounding box localization under cross-location conditions, rather than this robustness arising from the potato training data alone. The strong and location-invariant localization of the majority edible class is consistent with this interpretation. As no from-scratch (random initialization) ablation was performed, the relative contribution of COCO pretraining versus in-domain training cannot be isolated and remains a limitation of the present study.

Nevertheless, the external results also show that cross-location robustness is not uniform across all classes. The model remained highly reliable for edible potatoes, but performance decreased for feed and rotten classes. This confirms that location robustness should not be assessed only using aggregate metrics. Per-class analysis is necessary, especially when minority classes are more important for practical sorting decisions.

### 4.3. Class-Level Detection Challenges

The most important class-level limitation was the reduced performance for the feed and rotten classes. The edible class generalized almost without loss, with external F1 remaining close to the internal value. This is expected because edible potatoes represent the majority of the dataset and have more consistent visual characteristics.

The feed class showed the largest generalization drop. The main issue was not missed feed potatoes, but reduced precision caused by edible potatoes being classified as feed. This suggests that the model sometimes interpreted soil residue, mild discoloration, minor surface damage, or natural texture variation as quality defects. From a practical perspective, this type of error would lead to unnecessary rejection or downgrading of otherwise acceptable potatoes.

The rotten class showed a different error pattern. Precision remained relatively high, but recall decreased on the external dataset. This means that when the model predicted rotten, it was often correct, but some rotten potatoes were missed. This is more critical for sorting applications because rotten potatoes should be reliably removed from the product stream. The likely reason is that rot symptoms can be localized, partially hidden, or visible only from one side of the tuber.

These results show that the main challenge is not general potato detection, but fine-grained quality discrimination. The visual boundary between edible and feed is inherently subjective, while rotten detection depends strongly on defect visibility. This supports the need for additional data diversity, stricter annotation rules, and possibly multi-view imaging in future systems.

### 4.4. Implications for Real Sorting Systems

The results are encouraging for the development of a low-cost RGB-based optical sorting system. The proposed setup demonstrates that standard RGB imaging combined with YOLO-based detection can provide reliable localization and classification of multiple potatoes in conveyor-like scenes.

For real online sorting, however, several practical requirements must be considered. First, inference speed must be sufficient for the expected throughput of the sorting line. Second, model predictions must remain stable across different batches and farms. Third, minority classes such as feed and rotten must be detected with sufficient reliability, because these classes directly affect product quality and sorting decisions.

The high performance of yolo26_l suggests that the model is a strong candidate for further system integration. However, the observed feed and rotten errors indicate that a single-view RGB system may not be sufficient for fully reliable industrial deployment. In particular, rotten potatoes that are missed represent a practical risk if defects are located on the non-visible side of the tuber.

Future sorting systems may therefore benefit from combining object detection with additional mechanisms, such as multi-view imaging, temporal tracking across consecutive frames, class-specific confidence thresholds, or conservative rejection strategies. For example, the confidence threshold for rotten potatoes could be adjusted differently from the edible class if the goal is to reduce missed defective tubers.

### 4.5. Comparison with Related Work and Limitations of the Study

The results of the present study are broadly consistent with previous findings demonstrating the applicability of YOLO-based architectures for automated potato quality inspection. Li et al. [18] proposed an improved YOLOv5s model integrating coordinate attention and multi-scale feature fusion modules, achieving a precision of 82.0%, recall of 86.6%, F1-score of 84.3%, and mAP@0.5 of 85.1% across six defect categories under controlled single-location imaging conditions. Li et al. [19] extended this line of work with a multi-task YOLOv8s-based architecture capable of simultaneously performing edibility detection and surface defect segmentation, reporting a detection mAP@0.5 of 96.7% and recall of 92.3% on a single-location dataset. These results confirm that YOLO-based models can achieve strong performance when training and testing data originate from the same acquisition environment, which is consistent with the high internal performance observed in the present study, where all 25 evaluated configurations achieved mAP@0.5:0.95 above 0.863.

The present study also builds directly on prior work conducted within the same research group, in which Verk et al. evaluated Mask R-CNN for potato instance segmentation under comparable acquisition conditions using the same imaging platform and three-class quality taxonomy [30]. That study reported a best-case mAP of 0.878 and an F1-score of 0.597 at a processing speed of 6.46 FPS. The yolo26_l model evaluated in the present work surpasses these results on both the internal and external test sets while operating at 23.7 FPS (GPU compute), demonstrating that single-stage YOLO-based detectors offer a favorable alternative to two-stage architectures for this application in terms of both accuracy and inference throughput. Critically, the Mask R-CNN evaluation was conducted on a single-location dataset without cross-location testing, which further motivates the present study’s focus on generalization robustness as a primary evaluation criterion.

However, direct numerical comparison between these studies and the present work is methodologically limited. The referenced studies employ different class taxonomies—six fine-grained defect categories in [18] versus the three practical sorting classes used here—different imaging setups, and datasets collected from single acquisition sites without cross-location evaluation. High performance reported under such conditions does not necessarily reflect deployment robustness when the model is applied to potatoes from a different farm, harvesting batch, or production region. This limitation has been explicitly identified in the broader agricultural computer vision literature, where domain shift between training and deployment environments has been shown to cause substantial performance degradation in models that were not specifically designed or evaluated for cross-location generalization [23,24,26].

The main contribution of the present study relative to existing potato inspection work is therefore not the absolute level of detection accuracy, but the explicit evaluation of cross-location generalization under realistic agricultural variability. By training on the Kranj dataset and evaluating on the completely unseen Slovenj Gradec dataset, the experimental protocol reflects deployment conditions more faithfully than a standard random split. The best-performing yolo26_l configuration achieved an external F1-score of 0.9176 and mAP@0.5:0.95 of 0.8157, demonstrating that a degree of cross-location robustness is achievable with a standard YOLO architecture trained on a relatively modest dataset using a standard supervised protocol, with no domain adaptation or target-location fine-tuning applied in this study. A concurrent benchmark of YOLO architectures across multispectral vineyard domains similarly confirms that cross-dataset transfer performance and error-centric reliability metrics are more informative for deployment readiness than single-domain accuracy alone [28].

Several limitations of this study should be acknowledged. First, the dataset was collected at only two locations, which does not fully represent the variability expected across different farms, seasons, cultivars, and storage conditions. Second, the dataset exhibits class imbalance, with edible potatoes representing most of the annotated instances, which likely contributed to the weaker generalization observed for feed and rotten classes. Third, despite a standardized labeling protocol, borderline cases between edible and feed remain inherently subjective, and annotation variability across sites cannot be fully excluded. Fourth, the system relies on single-view RGB imaging, which limits the discriminative information available for defects localized on non-visible tuber surfaces. Finally, all experiments were conducted under controlled illumination and fixed acquisition geometry; real industrial environments may introduce additional sources of variability, including motion blur, mechanical vibration, and variable throughput conditions that were not represented in this evaluation.

Two further baseline categories remain outside the scope of the present study. First, classical computer-vision approaches (e.g., threshold- or color-histogram-based segmentation) were not implemented as quantitative baselines; while the deep-learning advantage over such methods is well established in the dense multi-instance setting considered here, a direct quantitative comparison was not performed. Second, no domain-adaptation baseline (e.g., fine-tuning of upper layers on a small sample of target-location data) was evaluated, so the reported cross-location performance should be read as a lower bound achievable without adaptation rather than as evidence that adaptation would not yield further gains. Both directions are natural extensions for future work.

The statistical analysis reported in Section 3.7 quantifies image-level variability: it establishes with high confidence that yolo26_l outperforms the other configurations across the external test images, using paired per-image comparisons on the fixed trained models. It does not quantify run-to-run variance arising from stochastic training effects, since each architecture was trained once with a single random seed (seed 42). Establishing that the architectural ranking itself is stable would require retraining each configuration multiple times with different seeds, which was beyond the computational scope of this benchmark. The present results should therefore be read as strong evidence that yolo26_l is superior on this external test set, but not as proof that this architecture would necessarily rank first under repeated retraining. Multi-seed repetition is identified as an important direction for future work.

### 4.6. Future Research Directions

Future work should focus on increasing dataset diversity and improving reliability for minority classes. Additional data should be collected from more farms, seasons, potato varieties, and contamination levels. This would allow a more complete evaluation of location and batch robustness.

A second direction is improving rotten and feed classification. This may include targeted data collection for minority classes, class-balanced training strategies, hard-example mining, and class-specific threshold optimization. More detailed annotation categories could also help separate cosmetic defects from severe quality degradation.

A third direction is the integration of multi-view or video-based inspection. Since some defects are visible only from specific angles, capturing multiple views of the same potato could improve rotten detection and reduce classification ambiguity. Temporal tracking could also help maintain consistent predictions as potatoes move through the sorting line.

Finally, the system should be evaluated in a real conveyor-based sorting prototype. Such testing should include inference speed, throughput, synchronization with actuation mechanisms, and robustness under continuous operation. Prior to physical deployment, discrete-event simulation of the sorting line—as applied to the optimization of automated sorting systems in other domains, where it has been used to identify throughput bottlenecks and synchronization constraints before hardware implementation [31]—could help model the interaction between detection latency and mechanical actuation. This would provide the next step from model-level validation toward practical deployment in affordable optical sorting systems for small and medium-scale farms.

## 5. Conclusions

This study investigated the feasibility of a low-cost RGB-based optical inspection system for automated potato quality detection using deep learning-based object detection. A controlled imaging platform was developed using commodity hardware, and a dataset of 19,805 manually annotated instances across 1361 images was collected from two geographically distinct farm locations in Slovenia. A systematic benchmark of 25 YOLO model configurations spanning five architecture families was conducted using a strict cross-location evaluation protocol, in which models were trained exclusively on data from one location and evaluated on a completely unseen dataset from a second location.

The results demonstrate that all evaluated YOLO configurations can reliably learn the three-class potato quality detection task under in-distribution conditions, with every model achieving F1 ≥ 0.906 on the internal test set. However, in-domain performance alone proved insufficient for model selection, as the ranking changed substantially under cross-location evaluation. Among all configurations, yolo26_l achieved the best cross-location performance, with an external F1-score of 0.918 and mAP@0.5:0.95 of 0.816, while also recording the smallest generalization drop of any evaluated model (ΔF1 = 0.029). These results suggest that YOLO26’s architectural innovations—including Progressive Loss Balancing, Small-Target-Aware Label Assignment, and the MuSGD optimizer—may contribute to more transferable visual representations under domain shift, though ablation studies would be required to confirm this hypothesis.

Per-class analysis revealed that generalization performance was not uniform across quality classes. The edible class transferred with minimal loss (ΔF1 = 0.002), while feed and rotten classes remained the primary bottleneck. Feed detection suffered primarily from excess false positives driven by edible-to-feed confusion, while rotten detection was impaired by increased false negatives—missed detections of tubers with localized or partially occluded decay symptoms. These distinct failure modes reflect the combined effect of class imbalance, visual ambiguity at class boundaries, and the inherent limitations of single-view RGB imaging for defect detection. Furthermore, the rotten class encompasses a wide range of biologically distinct decay types—including bacterial soft rot, late blight, dry rot, and fungal infections—each presenting different visual signatures. The training dataset did not systematically represent this diversity, which likely limited the model’s ability to generalize to rare or visually atypical decay patterns. Expanding future datasets to capture the full spectrum of rot manifestations will be essential for robust deployment in real sorting systems.

From a practical perspective, the results confirm that affordable RGB-based potato sorting systems are technically feasible and that a meaningful degree of cross-location robustness is achievable with standard supervised training alone, without any target-location adaptation in the present study. At the same time, the observed minority class errors indicate that full deployment readiness will require additional measures, including expanded multi-location datasets, class-balanced training strategies, and potentially multi-view imaging to address defects on non-visible tuber surfaces. Cross-location evaluation, as demonstrated in this study, should be considered a standard component of the validation protocol for any optical sorting system intended for deployment across diverse agricultural environments.

## Figures and Tables

**Table 1:** Overview of the potato image datasets used in this study.

Location	No. of Images	Role in Study	Avg. Potatoes per Image
Kranj	1060	Training, validation, and internal testing	12–15
Slovenj Gradec	301	External testing	12–15

**Table 2:** Distribution of annotated instances by quality class and acquisition location.

Class	Kranj	Slovenj Gradec	Total
Edible	9490	3819	13,309
Feed	3243	427	3670
Rotten	2577	249	2826
Total	15,310	4495	19,805

**Table 3:** Operational definition of annotation classes used in the dataset.

Class	Operational Definition	Inclusion Criteria
Edible	Marketable potato without major visible quality defects.	No severe visible damage, no advanced decay, visually acceptable for human consumption.
Feed	Non-marketable potato with visible defects, but without advanced decay.	Visible external defects, discoloration, surface damage, or reduced visual quality not classified as rot.
Rotten	Potato with clearly visible advanced decay.	Evident rotting symptoms such as strong discoloration, tissue breakdown, or severe surface deterioration.

**Table 4:** Data partitioning used for model development and evaluation.

Subset	Source Location	No. of Images	Proportion	Purpose
Training	Kranj	848	80%	Model training
Validation	Kranj	106	10%	Monitoring during training
Internal test	Kranj	106	10%	In-distribution evaluation
External test	Slovenj Gradec	301	100%	Cross-location evaluation

**Table 5:** Preprocessing and augmentation settings used in model training.

Operation	Setting	Applied to
Image resizing	1280 × 932 (no tiling)	Train/validation/internal test/external test
Normalization	Pixel values scaled to [0, 1] (framework default; no ImageNet mean/std subtraction)	Train/validation/internal test/external test
Horizontal flip	Probability 0.8	Training only
Translation	Fraction 0.10	Training only
HSV hue augmentation	Fraction 0.02	Training only
HSV saturation augmentation	Fraction 0.30	Training only
Mosaic, MixUp, CutMix, copy-paste, scale, rotation, shear, perspective, vertical flip, HSV value	Disabled	Training only
Additional eval-time preprocessing	None (deterministic resize + normalization only)	Validation/internal test/external test

**Table 6:** Common training configuration used for all evaluated models.

Parameter	Value
Frameworks	Ultralytics YOLO 8.4.18/PyTorch 2.5.1 (torchvision 0.20.1)
Epochs	150 (early stopping, patience 30)
Batch size	4
Input image size	1280 (native aspect ratio)
Initial learning rate	0.001
Optimizer	AdamW
Hardware	NVIDIA GeForce RTX 4090, 24 GB VRAM (NVIDIA Corporation, Santa Clara, CA, USA); AMD Ryzen 9 5950X, 16 cores/32 threads (Advanced Micro Devices, Santa Clara, CA, USA); 64 GB RAM; CUDA 12.1; NVIDIA driver 595.79
Weight initialization	COCO-pretrained (.pt checkpoints)
Random seed	42
Final learning rate fraction (lrf)	0.01
Learning rate scheduler	Linear (cos_lr = False)
Momentum/β_1_	0.937
Weight decay	0.0005
Warmup epochs	3.0
Warmup momentum	0.8
Warmup bias learning rate	0.1
Checkpoint selection metric	mAP@0.5:0.95 (validation)
Confidence threshold (evaluation)	0.25
NMS IoU threshold	0.7
Maximum detections per image	300

**Table 7:** Detection performance of all 25 YOLO configurations on the internal Kranj test set and the external Slovenj Gradec test set.

	Internal		External		Comparison	
Model	F1	mAP@0.5:0.95	F1	mAP@0.5:0.95	ΔF1	ΔmAP@0.5:0.95
yolov8_n	0.9164	0.8711	0.8192	0.7724	0.0972	0.0987
yolov8_s	0.9411	0.8803	0.8402	0.7889	0.1009	0.0914
yolov8_m	0.9438	0.8799	0.8662	0.7286	0.0776	0.1514
yolov8_l	0.9234	0.8793	0.8418	0.7392	0.0816	0.1400
yolov8_x	0.9430	0.8757	0.8362	0.7136	0.1068	0.1621
yolov9_n	0.9204	0.8657	0.7922	0.7548	0.1282	0.1109
yolov9_s	0.9483	0.8758	0.8746	0.7749	0.0737	0.1009
yolov9_m	0.9433	0.8779	0.8698	0.7749	0.0735	0.1031
yolov9_l	0.9348	0.8739	0.8815	0.7893	0.0532	0.0846
yolov9_x	0.9065	0.8631	0.8077	0.7637	0.0988	0.0994
yolov10_n	0.9360	0.8653	0.8501	0.7243	0.0860	0.1411
yolov10_s	0.9296	0.8658	0.8433	0.7055	0.0863	0.1603
yolov10_m	0.9409	0.8811	0.8610	0.7614	0.0799	0.1196
yolov10_l	0.9381	0.8740	0.8333	0.7457	0.1048	0.1283
yolov10_x	0.9418	0.8821	0.8341	0.7767	0.1076	0.1055
yolo11_n	0.9324	0.8681	0.8592	0.7352	0.0731	0.1330
yolo11_s	0.9517	0.8788	0.8638	0.7768	0.0879	0.1019
yolo11_m	0.9362	0.8763	0.8438	0.7479	0.0924	0.1284
yolo11_l	0.9374	0.8746	0.8598	0.7993	0.0776	0.0753
yolo11_x	0.9456	0.8796	0.8738	0.7510	0.0718	0.1286
yolo26_n	0.9107	0.8649	0.8448	0.7392	0.0659	0.1257
yolo26_s	0.9318	0.8686	0.8419	0.7141	0.0900	0.1545
yolo26_m	0.9404	0.8793	0.8430	0.7464	0.0974	0.1329
yolo26_l	0.9464	0.8895	0.9176	0.8157	0.0288	0.0738
yolo26_x	0.9261	0.8753	0.8459	0.7585	0.0801	0.1168

**Table 8:** Per-class performance of yolo26_l on the internal Kranj test set (106 images) and the external Slovenj Gradec test set (301 images). ΔF1 reports the per-class drop from internal to external.

	Internal		External		
Class	F1	AP@0.5:0.95	F1	AP@0.5:0.95	ΔF1
edible	0.9640	0.9239	0.9618	0.9848	0.0022
feed	0.8805	0.8618	0.6518	0.6375	0.2287
rotten	0.9638	0.8828	0.7773	0.8248	0.1865

**Table 9:** Absolute error counts for all 25 YOLO configurations on the internal Kranj (106 images) and external Slovenj Gradec (301 images) test sets. FP: false positives; FN: false negatives; FP/img and FN/img: per-image rates.

Model	Int. FP	Int. FN	Int. FP/Img	Int. FN/Img	Ext. FP	Ext. FN	Ext. FP/Img	Ext. FN/Img
yolov8_n	175	101	1.65	0.95	1088	622	3.61	2.07
yolov8_s	112	80	1.06	0.75	846	626	2.81	2.08
yolov8_m	106	77	1.00	0.73	719	512	2.39	1.70
yolov8_l	158	94	1.49	0.89	904	571	3.00	1.90
yolov8_x	101	84	0.95	0.79	822	675	2.73	2.24
yolov9_n	157	104	1.48	0.98	1151	792	3.82	2.63
yolov9_s	96	72	0.91	0.68	655	493	2.18	1.64
yolov9_m	110	75	1.04	0.71	687	507	2.28	1.68
yolov9_l	126	87	1.19	0.82	612	470	2.03	1.56
yolov9_x	194	115	1.83	1.08	1174	655	3.90	2.18
yolov10_n	125	84	1.18	0.79	793	586	2.63	1.95
yolov10_s	144	87	1.36	0.82	938	534	3.12	1.77
yolov10_m	116	77	1.09	0.73	730	545	2.43	1.81
yolov10_l	111	90	1.05	0.85	859	671	2.85	2.23
yolov10_x	113	77	1.07	0.73	885	646	2.94	2.15
yolo11_n	131	90	1.24	0.85	773	527	2.57	1.75
yolo11_s	90	67	0.85	0.63	707	540	2.35	1.79
yolo11_m	129	80	1.22	0.75	870	580	2.89	1.93
yolo11_l	118	86	1.11	0.81	717	565	2.38	1.88
yolo11_x	102	75	0.96	0.71	646	506	2.15	1.68
yolo26_n	204	94	1.92	0.89	934	525	3.10	1.74
yolo26_s	134	89	1.26	0.84	917	561	3.05	1.86
yolo26_m	120	75	1.13	0.71	889	572	2.95	1.90
yolo26_l	107	68	1.01	0.64	462	293	1.53	0.97
yolo26_x	145	97	1.37	0.92	861	569	2.86	1.89

**Table 10:** Inference latency (GPU compute only) and model complexity for all 25 YOLO configurations.

Model	Int. Lat. (ms)	Int. FPS	Params (M)	FLOPs (G)
yolov8_n	22.9	43.7	3.0	32.3
yolov8_s	27.6	36.2	11.1	113.8
yolov8_m	38.9	25.7	25.8	314.8
yolov8_l	38.9	25.7	43.6	659.3
yolov8_x	43.9	22.8	68.1	1029.6
yolov9_n	36.9	27.1	2.0	30.4
yolov9_s	42.8	23.3	7.2	106.9
yolov9_m	43.6	22.9	20.0	306.1
yolov9_l	45.6	21.9	25.3	409.3
yolov9_x	48.4	20.6	57.4	756.6
yolov10_n	18.8	53.1	2.3	26.1
yolov10_s	29.4	34.1	7.2	85.7
yolov10_m	37.6	26.6	15.3	235.4
yolov10_l	40.7	24.6	24.3	480.1
yolov10_x	41.2	24.2	29.4	639.9
yolo11_n	23.4	42.8	2.6	25.3
yolo11_s	32.2	31.1	9.4	85.2
yolo11_m	35.1	28.5	20.0	270.6
yolo11_l	45.8	21.9	25.3	346.3
yolo11_x	37.5	26.6	56.8	777.7
yolo26_n	22.1	45.2	2.4	20.8
yolo26_s	34.8	28.7	9.5	82.1
yolo26_m	38.8	25.8	20.4	271.4
yolo26_l	42.2	23.7	24.7	344.4
yolo26_x	37.9	26.4	55.6	773.5

**Table 11:** Segmented latency profiling (mean across 25 models): preprocessing, forward pass, and NMS for both test sets.

Stage	Internal Test	External Test
Preprocessing (incl. disk I/O)	104.8 ms	105.2 ms
Forward pass	49.2 ms	48.7 ms
NMS	1.00 ms	1.04 ms

**Table 12:** Comparison of the best YOLO configuration (yolo26_l) and the Faster R-CNN ResNet-50 FPN baseline on the internal and external test sets.

Model	Int. F1	Int. mAP@0.5:0.95	Ext. F1	Ext. mAP@0.5:0.95
yolo26_l	0.9464	0.8895	0.9176	0.8157
yolov9_l	0.9348	0.8739	0.8815	0.7893
Faster R-CNN ResNet-50 FPN	0.9211	0.8544	0.8805	0.7876

**Table 13:** Top-five YOLO configurations by mean per-image external-test F1, with bootstrap 95% confidence intervals (10,000 resamples).

Rank	Model	Mean per-Image External F1	95% CI
1	yolo26_l	0.9318	[0.9244, 0.9390]
2	yolov9_l	0.8969	[0.8885, 0.9049]
3	yolov9_m	0.8923	[0.8839, 0.9003]
4	yolov8_m	0.8920	[0.8837, 0.9003]
5	yolo11_x	0.8898	[0.8811, 0.8984]

## Data Availability

The datasets generated and analyzed during the current study are available from the corresponding author upon reasonable request.

## Supplementary Materials

The following supporting information can be downloaded at: https://www.mdpi.com/article/10.3390/foods15122121/s1, Table S1: Complete training configuration (key args.yaml fields) for all 25 YOLO runs; Table S2: Per-epoch training/validation metrics for all 25 configurations, provided in XLSX format and as an auxiliary machine-readable CSV copy; Figure S1: Training convergence curves (validation mAP@0.5:0.95) for all 25 configurations.
