# Supplementary figures and images for: OPTIFARM: Benchmarking YOLO Architectures for Location-Robust Potato Quality Detection
Source: Foods. 2026 Jun 12;15(12):2121. doi: 10.3390/foods15122121 (PMC13298113; doi:10.3390/foods15122121)

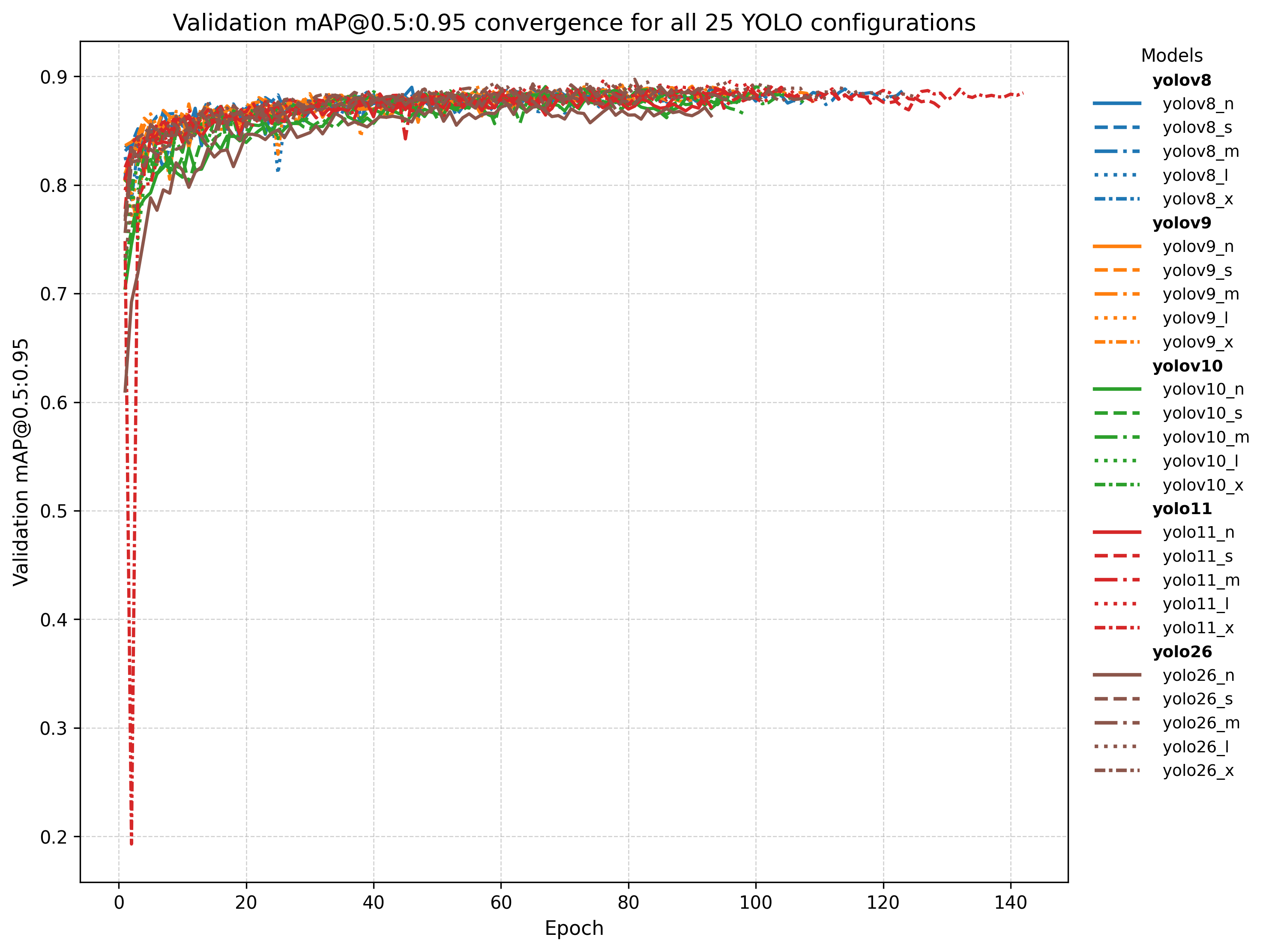

Supplement: Supplementary file 1 [file foods-15-02121-s001.zip › Figure_S1_convergence_all25.png]
